# Assessing Creatine-Related Gene Expression in Kidney Disease: Can Available Data Give Insights into an Old Discussion?

**DOI:** 10.3390/nu17040651

**Published:** 2025-02-12

**Authors:** Matheus Anselmo Medeiros, Bento João Abreu, João Paulo Matos Santos Lima

**Affiliations:** 1 Bioinformatics Graduate Program, Digital Metropolis Institute, Federal University of Rio Grande do Norte (UFRN), Natal 59078-400, RN, Brazil; matheus.medeiros.060@ufrn.edu.br; 2 Department of Morphology, Biosciences Center, Federal University of Rio Grande do Norte (UFRN), Natal 59078-970, RN, Brazil; abreubj@gmail.com; 3 Bioinformatics Multidisciplinary Environment (BioME), Digital Metropolis Institute (IMD), Federal University of Rio Grande do Norte (UFRN), Natal 59078-970, RN, Brazil

**Keywords:** creatine monohydrate, kidney, renal injury, review

## Abstract

The impact of creatine supplementation on individuals with kidney disease or pathological conditions with an increased risk of developing kidney dysfunction remains an active discussion. However, the literature on gene expression related to cellular creatine uptake and metabolism under altered renal function is scarce. Therefore, the present study utilized comprehensive bioinformatics analysis to evaluate the expression of creatine-related genes and to establish their relationships to normal and disturbed renal conditions. We identified 44 genes modulated explicitly in response to creatine exposure from a gene enrichment analysis, including IGF1, SLC2A4, and various creatine kinase genes. The analysis revealed associations with metabolic processes such as amino acid metabolism, indicating a connection between creatine and tissue physiology. Using the Genotype-Tissue Expression Portal, we evaluated their basal tissue-specific expression patterns in kidney and pancreas tissues. Then, we selected several pieces of Gene Expression Omnibus (GEO) transcriptomic data, estimated their expression values, and established relationships to the creatine metabolism pathways and regulation, shedding light on the potential regulatory roles of creatine in cellular processes during kidney diseases. These observations also highlight the connection between creatine and tissue physiology, emphasizing the importance of understanding the balance between endogenous creatine synthesis and creatine uptake, particularly the roles of genes such as GATM, GAMT, SLC6A8, and IGF1, under several kidney dysfunction conditions. Overall, the available data in the biological databases can provide new insights and directions into creatine’s effects and role in renal function.

## 1. Introduction

Creatine, a naturally occurring compound primarily found in muscle tissue, has garnered significant attention in sports nutrition and clinical research due to its potential benefits in enhancing physical performance and supporting various health conditions [1,2]. Traditionally recognized for its role in energy metabolism, creatine supplementation has been widely adopted by athletes and fitness enthusiasts to enhance exercise capacity and recovery. Furthermore, creatine supplementation may benefit a range of acute and chronic clinical conditions [3]. It is currently associated with positive effects on heart disease, muscle dystrophies [4], and brain health, such as cognitive processing, brain function, memory, and trauma recovery [5,6,7]. However, its implications for renal function and safety in individuals with pre-existing kidney conditions remain less well understood.

The continuous, low-grade, and non-enzymatic degradation of creatine produces creatinine as the resultant end product [8]. After filtration through the renal glomeruli, creatinine and plasma creatine are excreted. To counterbalance the daily depletion, the classic understanding is that our body synthesizes creatine through two reactions: the first occurs in the kidneys, where the enzyme arginine amidinotransferase (GATM/AGAT) converts arginine and glycine into guanidinoacetate, whereas the second reaction involves guanidinoacetate N-methyltransferase (GAMT), which catalyzes the conversion of guanidinoacetate to creatine through methylation in the liver [9]. However, this notion has been intensely debated since several gene expression studies have shown the expression of both GATM and GAMT in other tissues [10] and significant contributions from other tissues may be common [11].

On the other hand, it is well known that the serum creatinine concentration is a crude index of renal function [8]. Nevertheless, exogenous creatine intake markedly increases urine creatinine concentration and excretion, potentially leading to minor changes in markers of renal and hepatic function [12], which leads to the presumption that creatine supplementation could pose risks to healthy individuals, despite the lack of concrete evidence suggesting the opposite. The first concern about adverse effects from creatine supplementation emerged after two works in the late 1990s [13,14]. For instance, Pritchard and Kalra reported a case where a patient with previously controlled renal lesions experienced changes in kidney function after creatine supplementation. Notably, these alterations were ameliorated upon cessation of the supplementation regimen [14]. Since then, concerns have arisen about creatine’s potential impact on renal function, despite increasing scientific interest.

The association between creatine and renal injuries primarily originates from its tendency to elevate serum creatinine levels [4]. However, the interpretation of serum creatinine levels in the context of creatine supplementation must be cautious, as creatine can lead to false-positive results for renal injury due to non-enzymatic hydrolysis generating creatinine.

Recent advancements in bioinformatics have provided new opportunities to explore the molecular mechanisms underlying creatine’s impact on renal tissues. By integrating large-scale genomic data and utilizing advanced analytical tools, researchers can elucidate the interactions between creatine and renal cellular processes, including hypertrophy, apoptosis, and repair mechanisms [15,16,17]. This approach not only enhances our understanding of creatine’s biological effects but also facilitates the identification of potential molecular markers indicative of kidney health and function.

Although a substantial body of evidence supports the safety of creatine supplementation in healthy individuals, concerns about its effects on populations with renal impairment persist [18]. In fact, the literature presents conflicting findings: some studies suggest that creatine may slow the progression of kidney disease, while others report potential adverse effects [19,20]. To address these uncertainties, this study employs bioinformatics analyses to explore the molecular interactions and pathways influenced by creatine-related gene expression in renal conditions, aiming to clarify its impact on kidney structure and function.

## 2. Methods

We searched for “creatine” in the Stitch Database [21], focusing on *Homo sapiens* to explore known and predicted interactions between chemicals and proteins. Then, using the advanced settings, we restricted the search to the integration of RNA-Seq expression data from kidney tissues using the Human Protein Atlas database. In the updated gene list, the proteins AKT1, AKT2, and AKT3 appeared as tissue-specific interactions. We also used the restriction from the Tissues Database, using “kidney” as a search term.

To widen our search for creatine-related genes, we also used the Comparative Toxicogenomics Database (CTD) [22], using “creatine” as a search term. After obtaining a list of genes from CTD, both gene lists from CTD and Stitch databases were submitted to a batch query in the CTD to retrieve the gene–disease interactions. Then, we merged all the gene terms into one list. From this merged list, we performed a gene enrichment analysis (GEA), using enrichR [23] and enrich-Kg [24] to explore the biological functions and metabolic pathways of the identified genes, using Gene Ontology (GO) and the Kyoto Encyclopedia of Genes and Genomes (KEGG), respectively. We also performed a GEA against ReactomeDB. We retrieved the general expression panel of these genes for kidney tissues from the GTEx Portal (https://gtexportal.org, accessed on 11 December 2024). Additionally, we conducted a GEA against the kinase regulation datasets ARCHS4_Kinases [25] and the Kinase Library 2023 [26], to assess the overall regulation of proteins encoded by the creatine-related genes in our list. The inclusion criteria were consistent across all analyses: selecting the first ten results based on a *p*-value cutoff of ≤0.05.

We also evaluated the expression of creatine-related genes using datasets and gene expression series from the Gene Expression Omnibus (GEO) database [27]. We used each gene symbol as the primary search term, applying the following criteria: (i) organism: *Homo sapiens*; (ii) differential expression: Up/down genes; (iii) dataset keywords: *Kidney* and *Renal tissues*. The exclusion criteria were undetected expression of the genes, experimental designs that did not compare disease/metabolic/chemical treatment conditions, and mutant cell lines. The gene expression series were analyzed using the R packages GEO2R (https://www.ncbi.nlm.nih.gov/geo/geo2r/, accessed on 11 December 2024), GEOquery [28], limma [29], and DESeq2 [30], employing a case–control approach. We considered significant differential expression (DE) expression values with Log_2_ Fold Change > 0.5 and *p*-Value > 0.05. Afterward, we generated specific plots for the evaluated genes using ggplot2 [30] and the Python programming language modules numpy [31] and matplotlib [32]. The scripts used are available on https://github.com/evomol-lab.

## 3. Results and Discussion

### 3.1. Creatine-Related Proteins on Stitch Database

**Figure 1:**
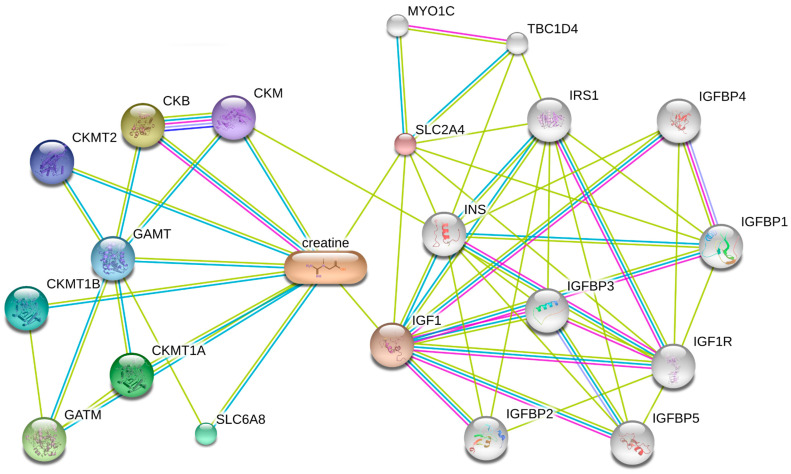
Chemical–protein interaction network of creatine obtained by STITCH (http://stitch.embl.de, accessed on 26 April 2024). Small nodes: protein of unknown 3D structure. Large nodes: protein 3D structure is known or predicted. Colored nodes: first shell of interactors. White nodes: second shell of interactors. Edges color represent interactions sources. Magenta: from curated databases; Pink: experimentally determined; Green, red and dark blue: predicted interactions from gene neighborhood, gene fusions and gene co-occurrence, respectively; Light green: predicted from text mining.

When searching for creatine in the Stitch Database, we identified the network of proteins with which creatine interacts. The SLC2A4 gene encodes the GLUT-4 protein itself, and the MYO1C gene encodes unconventional myosin-Ic responsible for the translocation of GLUT-4 from the cytoplasm to the cell membrane. Although there have been no assays observing increased expression of these proteins with creatine supplementation, this analysis indicates a strong interaction (score > 0.700) between creatine-SLC2A4 and SLC2A4-MYO1C (score > 0.700), as demonstrated in Figure 1. Despite these promising results, non-diabetic animals supplemented with creatine were diagnosed with pancreatitis and presented renal tubular necrosis [33]. Therefore, creatine supplementation should be further investigated in various nephropathies to assess the safety of its prescription.

We have also identified the presence of Insulin-like Growth Factor 1 (IGF1), which serves as a pivotal connector among proteins, including enzymes and transporters, that are directly involved in creatine metabolism and uptake, as well as other proteins and transcription factors (Figure 1). IGF1 plays a significant role in muscle development and metabolism, and it is intricately associated with muscle mass, strength development, and the proliferative capacity of muscle satellite cells. It is primarily produced by liver hepatocytes stimulated by growth hormone [34]. Given the structural and functional similarities between IGF1/IGF1R and insulin receptor signaling, IGF1’s influence extends beyond creatine metabolism to encompass broader metabolic functions [35]. IGF1 is also synthesized in multiple locations within the kidney, including mesangial cells, which are central to the development of kidney disease, with enhanced IGF1 signaling observed in various disease states, including diabetes [36]. In addition, altered IGF1 levels are linked to cardiovascular and renal diseases [37]. These observations underscore the multifaceted role of IGF1 in both creatine handling and general metabolic processes. The final gene list from the Stitch database included the following genes: SLC2A4, IGF1, GATM, SLC6A8, AKT1, AKT2, AKT3, PSMB5, PSMD3, SGK1, and PRPS1.

### 3.2. Comparative Toxicogenomics Database

We utilized the Comparative Toxicogenomics Database to analyze the effects of creatine on renal and pancreatic tissues. We explored the data to identify genes and pathways affected by creatine exposure and potential associations with specific physiological and metabolic processes. We observed that creatine exposure is associated with changes in the expression of 44 specific genes. After filtering the repetitive entries and excluding the pseudogenes, we obtained the following gene list from CTD: CKB, CKM, CKMT1A, CKMT1B, CKMT2, SLC16A12, and SLC6A8.

**Figure 2:**
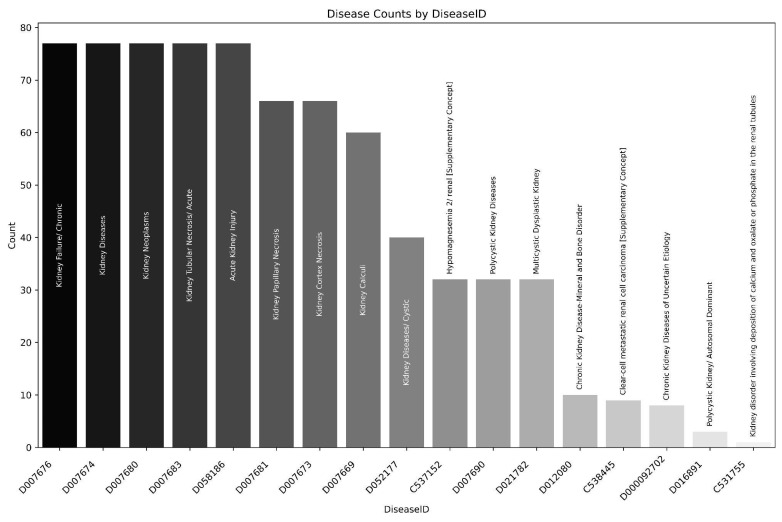
Number of gene–DiseaseID (MESH—Medical Subject Headings) associations from the creatine-related genes from Stitch and CTD databases. Names on bars indicate the Medical Subject Headings associated with each DiseaseID.

Using these two lists, we filtered the inferred gene–disease associations since the curated ones were enriched with genetic diseases (inborn metabolic errors), and filtered the results using the presence of the terms “kidney” and “renal”, obtaining 2596 and 818 rows for the Stitch and CTD gene lists, respectively. The higher number of entries in the Stitch list is probably associated with the initial filtering process for expression in kidney tissues. Figure 2 describes the DiseaseIDs (MESH) and counts from association with both gene lists. This analysis revealed that several diseases with similar names or related conditions, such as “Kidney Failure/Chronic”, “Kidney Diseases”, and “Kidney Neoplasms”, have identical counts, which may suggest potential clustering or co-occurrence of related diseases.

We found at least five disease associations for each gene, demonstrating that these genes presented altered expression or direct associations to kidney-related disorders, including chronic kidney diseases (CKDs), cancer, and kidney failure.

### 3.3. Gene Expression Analysis and Enrichment of Creatine-Related Genes

**Figure 3:**
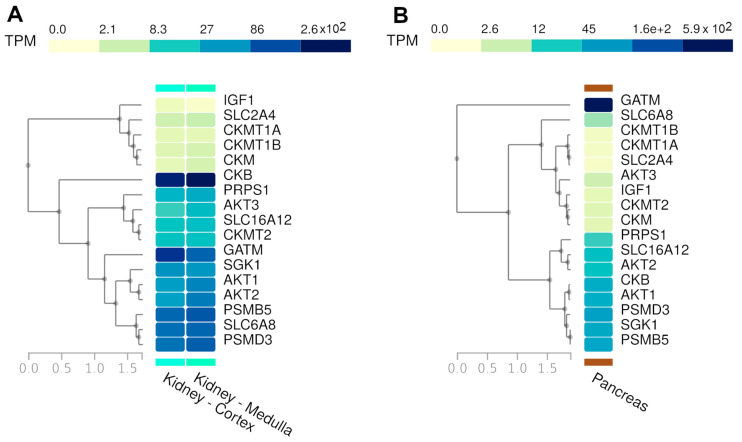
(**A**) Kidney and (**B**) pancreas tissue expression levels of the 17 creatine-related genes used in the present study (TPM—transcripts per million bases).

We merged the two gene lists above into one list (Table 1). Then, we obtained a comprehensive expression profile of these genes across all tissues using the Multi-Gene Query tool available on the GTEx Portal. Specifically, we highlighted the expression patterns in the kidney (Figure 3A) and pancreas tissues (Figure 3B).

We can observe similar expression patterns for several creatine-related genes, especially GATM, SLC6A12, and SLC6A8. These genes are responsible for cell creatine homeostasis, balancing the production and uptake of this compound [38]. GATM encodes the enzyme Glycine Amidinotransferase, the primary regulatory step in creatine biosynthesis, and the SLC6A8 and SLC6A12 genes encode the specific creatine transporter and the monocarboxylate transporter 12 (MCT12), respectively. The creatine concentration controls its synthesis by a feedback loop, regulating GATM expression [39].

**Table 1:** Creatine-related gene list according to our search criteria. Short gene descriptions were retrieved from GeneCards database [39].

Gene Symbol (*Homo sapiens*, txid:9606)	Short Description	Function and Previously Reported Relationship to Creatine Metabolism
SLC2A4	Insulin-regulated facilitative glucose transporter GLUT4.	This gene encodes an insulin-regulated facilitative glucose transporter, which was previously reported as induced by creatine supplementation.
IGF1	Insulin-like growth factor 1, a hormone involved in growth and development.	Structurally and functionally related to insulin, this protein acts by enhancing glucose uptake and glycogen synthesis in some tissues. The relationship to creatine metabolism and to the GLUT4 glucose transporter was obtained by mining text databases (StringDB).
GATM	Glycine amidinotransferase, key in creatine biosynthesis.	Encodes an enzyme that catalyzes the first reaction of endogenous creatine synthesis, converting arginine and glycine into guanidinoacetate.
GATM	Guanidinoacetate N-Methyltransferase, converts GAA in creatine.	Encodes an enzyme that catalyzes the conversion of guanidinoacetate to creatine through methylation, the second reaction of endogenous creatine synthesis.
SLC6A8	Creatine transporter involved in transporting creatine into cells.	As Solute Carrier Family 6 Member 8, this Na^+^ symport protein is specific to creatine transport into and out of the cells. In addition to creatine uptake, this transporter is also related to the nuclear receptor meta-pathway.
AKT1	AKT serine-threonine protein kinase 1, involved in signaling pathways regulating cell growth and survival.	Also referred to as protein kinase B alpha, beta, and gamma, members of the AKT family are responsible for the regulation of glucose uptake by mediating insulin-induced translocation of the SLC2A4/GLUT4 glucose transporter to the cell surface. Phosphorylation of PTPN1 at ‘Ser-50’ negatively modulates its phosphatase activity, preventing dephosphorylation of the insulin receptor and the attenuation of insulin signaling. There are more than 100 known substrates for these kinases, but no isoform specificity has been reported (GeneCards).
AKT2	AKT serine-threonine protein kinase 2, involved in metabolism and insulin signaling.	
AKT3	AKT serine-threonine protein kinase 3, involved in brain development and function.	
PSMB5	A component of the 20S core proteasome complex involved in protein degradation.	Both genes encode proteins of the complex responsible for degrading misfolded or damaged proteins within cells. Protein degradation by proteasomes is ATP-dependent and involves the ubiquitin-proteasome pathway, which is crucial for maintaining cellular homeostasis. The regulation of protein turnover by the proteasome could impact the availability of amino acids for creatine synthesis.
PSMD3	A non-ATPase subunit of the 26S proteasome involved in protein degradation.	
SGK1	Serum/glucocorticoid regulated kinase 1, involved in cellular stress response.	This gene encodes a serine/threonine-protein kinase, which is involved in the regulation of a wide variety of ion channels, membrane transporters, cellular enzymes, transcription factors, etc. It is involved in salt sensitivity of peripheral glucose uptake and upregulates Na^+^-mediated transporters, like the creatine transporter SLC6A8, and phosphorylates SLC2A4/GLUT4, upregulating its activity.
PRPS1	Phosphoribosyl pyrophosphate synthetase 1, involved in purine metabolism and nucleotide biosynthesis.	The enzyme PRPS1 is crucial for the synthesis of phosphoribosyl pyrophosphate (PRPP), which is an essential precursor for the biosynthesis of nucleotides. PRPS1 also plays a significant role in the pentose phosphate pathway, which is involved in cellular metabolism and energy production (GeneCards).
CKB	Creatine kinase B, which plays a role in energy homeostasis by transferring phosphate between ATP and various phosphogens.	The encoded cytoplasmic protein reversibly catalyzes phosphate transfer between ATP and creatine phosphate. Its activity is observed in the brain and other tissues.
CKM	Creatine kinase M, involved in energy homeostasis	CKM is an important serum marker for myocardial infarction and, as with other creatine kinases, reversibly catalyzes phosphate transfer between ATP and creatine phosphate. It is a paralog of CKB.
CKMT1A	Ubiquitous mitochondrial creatine kinase, involved in energy transduction and impaired in various diseases.	Both encoded proteins belong to the creatine kinase isoenzyme family, with mitochondrial-specific expression. They are responsible for the transfer of high-energy phosphate from mitochondria to creatine.
CKMT1B	Identical protein to CKMT1A and is also involved in energy transduction in tissues with large energy demands.	
CKMT2	Sarcomeric mitochondrial creatine kinase, involved in energy transduction in muscle tissues.	The encoded protein is an isoenzyme of ubiquitous CKMT, with sarcomere-specific expression.
SLC16A12	Transporter likely involved in monocarboxylic acid transport and associated with juvenile cataracts and renal glucosuria.	Protein related to amino acid transport pathways. It mediates cellular uptake of betaine and GABA in a sodium- and chloride-dependent process, and it is involved in renal and hepatic osmotic regulation.

**Figure 4:**
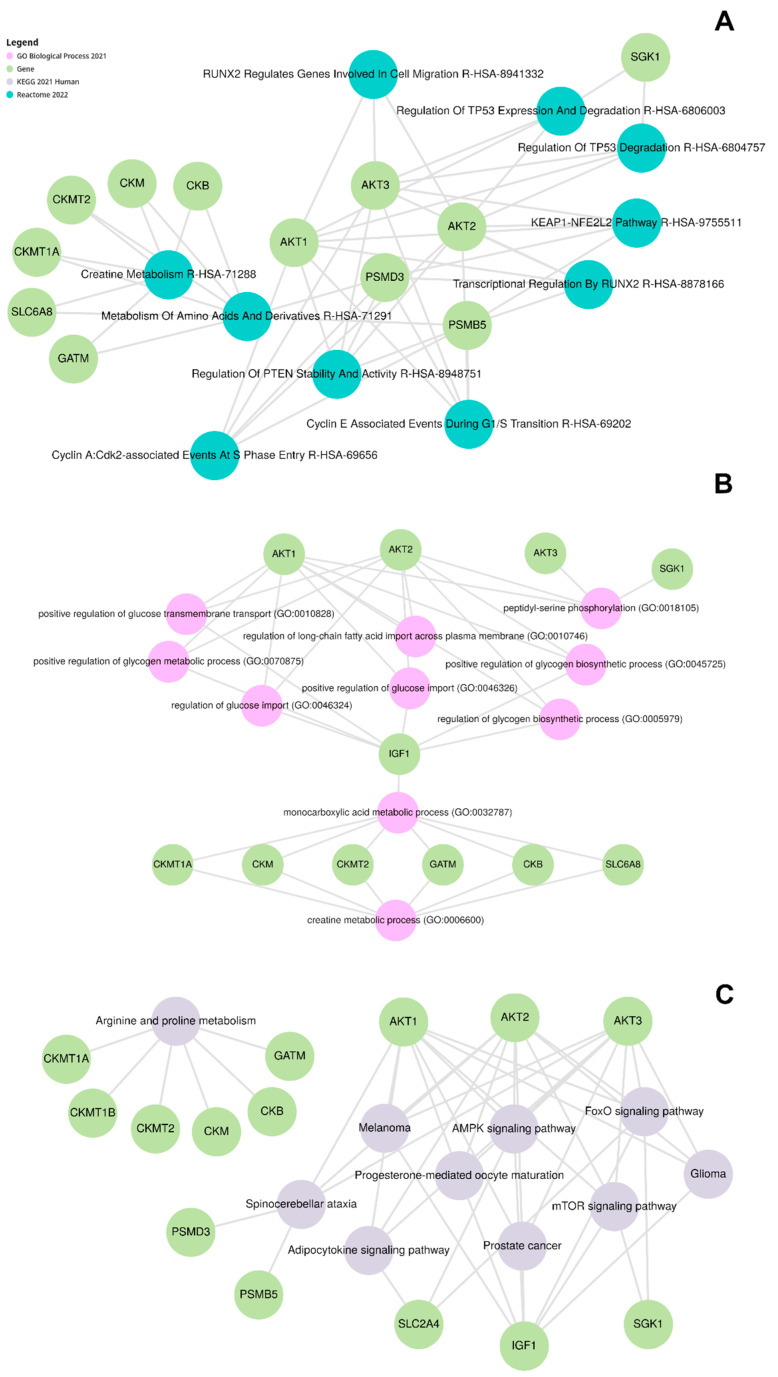
Gene Enrichment Analysis from EnrichR-Kg using the creatine-related gene list against the following databases: (**A**) ReactomeDB; (**B**) Gene Ontology; and (**C**) KEGG terms. Green circles represent the genes. The first ten results are represented, using *p*-values ≤ 0.05 as the threshold.

As anticipated, the first two terms from the gene enrichment analysis against the ReactomeDB were directly related to creatine and amino acid metabolism (Figure 4A). Interestingly, the remaining eight terms are associated with processes related to cell cycle regulation, including the degradation and stability of well-known tumor suppressor genes TP53 and PTEN. We also noted that several terms overlapped with the genes PSMB5, AKT2, AKT1, AKT3, PSMD3, and SGK1. This integration of amino acid metabolism with specific glycolysis reactions may explain their association with cell cycle control. For instance, the glycolytic enzyme PKM2 phosphorylates and activates ERK1/2, crucial for cell proliferation. Another glycolytic enzyme, PFKFB3, promotes cell proliferation by enhancing glycolytic ATP production and modulating the expression of cell cycle regulators [40]. The involvement of creatine kinase (CK) in mitosis regulation is well documented in the scientific literature. Moreover, previous studies have reported conflicting roles of creatine metabolism in tumor cells, with both upregulation and downregulation of CK potentially affecting cell viability and inducing cell death, depending on the tumor’s nature [41]. Through the GO analysis (Figure 4B), the study was able to categorize the genes in terms of biological processes, cellular components, and molecular functions, providing a broader view of these genes’ capabilities and actions, and how their expression may be influenced by the presence of creatine in renal tissues. Our first analysis was against the GO dataset to verify the terms associated with these 17 genes. The resulting network from the top ten *p*-value-rated results is described in Figure 4B. The only direct link between creatine metabolism and all the other processes is the gene IGF1, which also participates in several carbohydrate-metabolism-related processes. Increasing the number of results to the top 30 rated results did not retrieve any other gene links between creatine metabolism and other terms.

The pathway analysis using KEGG (Figure 4C) enabled the mapping of genes into specific metabolic pathway contexts, such as those involved in amino acid metabolism, elucidating how the convergence of metabolic activities and creatine supplementation may interact to impact renal function and homeostasis. Essentially, these analyses provided insights into how creatine can precisely alter renal metabolic pathways and which cellular processes may be particularly involved or affected. Additional findings are associated with insulin resistance and signaling metabolism, renal cell carcinoma, type 2 DM, central carbon metabolism in cancer, and others. The significant presence of AKT genes in various metabolic processes accounts for the numerous associated terms.

Moreover, this phase of analysis facilitated the identification of changes in the activity of specific genes that may be associated with processes such as cellular hypertrophy, apoptosis, and cellular repair. These findings are crucial for building a more comprehensive understanding of the implications of creatine intake, as alterations in cellular activity may have direct implications on renal function.

### 3.4. Pathway Analysis and Regulation by Kinases and Phosphatases

**Figure 5:**
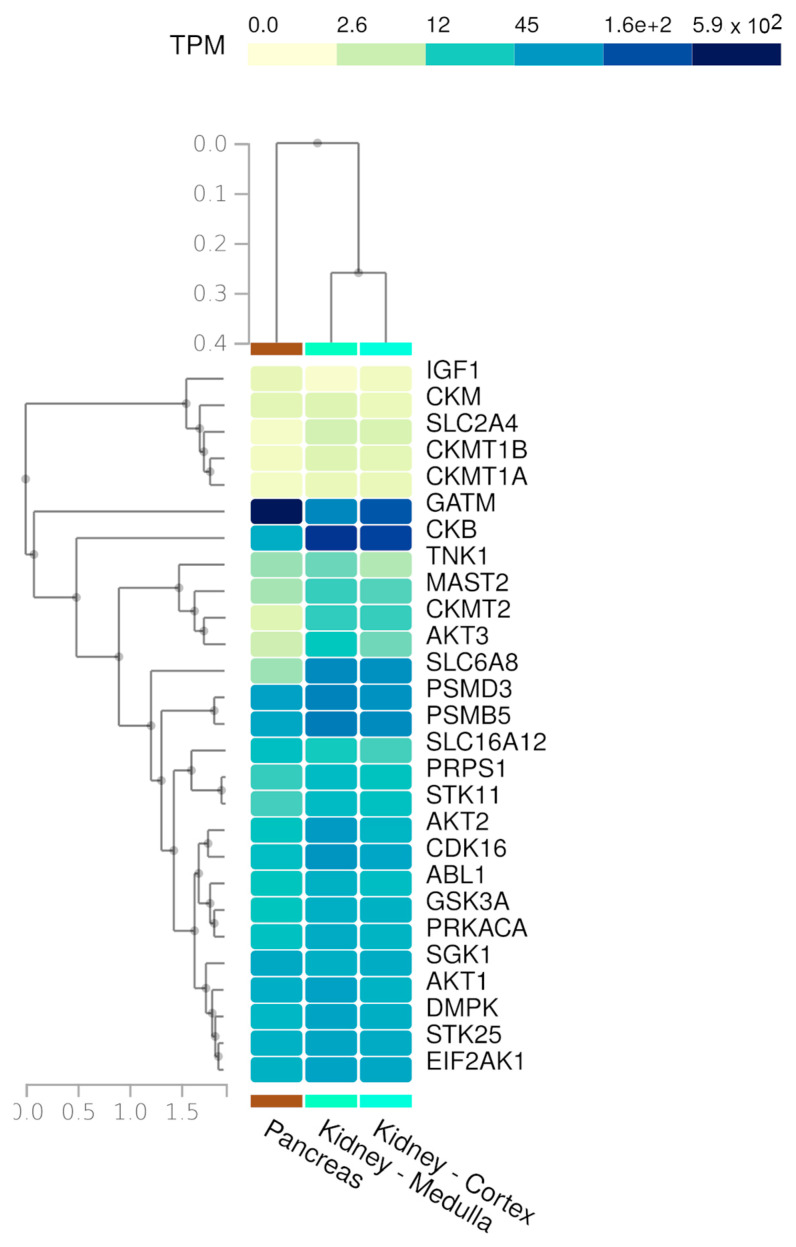
Expression of creatine-related genes and the kinases retrieved from gene enrichment analysis against kinase-specific databases (TPM—transcripts per million bases).

To investigate how kinase regulation processes influence creatine metabolism pathways, we performed a GEA of two kinase-specific databases: ARCHS4 and The Kinase Library 2023. After merging the results, we found 24 kinases that are directly or indirectly related to the creatine-related genes (Supplementary Table S1). We also joined this list with that of creatine genes to evaluate their normal expression in kidney and pancreas tissues from GTEx (Figure 5). All the kinases presented high basal expression in the two tested tissues. A noteworthy observation is that both tissues present a very similar expression of these genes, and are consequently grouped in the same cluster when we analyze them together with the expression of all other human tissues (Supplementary Figure S1).

The study of metabolic pathways and enzymatic regulation helps elucidate not only how creatine is metabolized by the kidneys, but also how this metabolism may affect other cellular functions through kinase modulation. For instance, the impact of creatine on cellular energy levels and renal tissue homeostasis may be mediated by pathways regulated by specific kinases, providing a direct link between creatine supplementation and renal health status [42]. Additionally, exploring kinase regulation opens pathways to understanding broader cellular events, such as signal transduction and cell cycle regulation, which may have significant implications in renal disease contexts or under conditions of oxidative stress.

We focused on the AKT gene family due to their significant role in intracellular signaling and the regulation of processes such as cell growth, proliferation, and survival [43]. AKT proteins are involved in several important metabolic pathways and are known to be sensitive to variations in nutrient levels, including creatine [44]. Therefore, understanding how creatine influences AKT activity can reveal significant insights into the molecular mechanisms that result in the physiological effects of creatine in the kidneys. For instance, changes in AKT activity may impact cellular energy balance and protection against oxidative stress. These findings are significant as they offer potential for therapeutic manipulation in conditions where renal function is compromised or where regulation of energy metabolism is crucial.

Furthermore, by studying the expression of these kinases and their potential regulation in different kidney conditions/diseases, we can highlight a new layer of complexity in understanding the effects of creatine. It is not just a supplement that can affect energy capacity and muscle performance, but also an agent that interacts with signaling mechanisms and cellular regulation that are pivotal for normal renal health and function.

### 3.5. GEO Dataset Analyses

We did not find specific datasets from human organisms or cell models that evaluated the creatine treatment or supplementation on renal tissues. Therefore, we restricted our search to datasets that evaluated specific kidney diseases to analyze the expression of the genes in our list. The first part was to search the list’s gene symbols against the GEO profile database, which uses curated datasets to evaluate individual gene expression profiles. Within our search criteria, the gene GATM presented the highest number of profiles, with 16 distinct ones, most of them with significant differential expression between the conditions tested. Then, we chose four distinct expression datasets from kidney conditions to widen the creatine-related gene assessment from the array (three) and RNA-Seq platforms (one).

**Figure 6:**
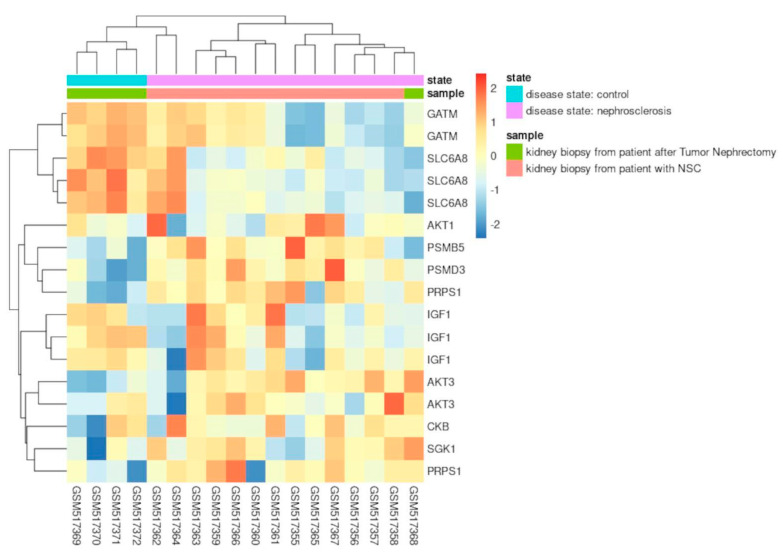
Heatmap with the expression of creatine-related genes, from the study of Neusser et al. [45] (GEO Accession number: GDS3712). The sample codes and gene symbols are represented horizontally and vertically, respectively. Sample states (TN—control; NSC—nephrosclerosis) are represented in the first colored line. The following cutoffs were used: *p*-values < 0.05 and Log_2_Fold Change ± 1.

The first dataset evaluated was from the study of Neusser et al. [45], which analyzed the genome-wide array expression of renal biopsy specimens from 14 patients with nephrosclerosis (NSC) against four tumor-free kidney specimens from patients undergoing tumor nephrectomy (TN) (GDS3712, GSE20602). The authors revealed the significant regulation of hypoxia-associated biological processes in the NSC samples, including angiogenesis, fibrosis, and inflammation. The glomerular expression levels of most genes regulated by hypoxia-inducible factors (HIFs) were also significantly altered in these samples [45]. We re-analyzed the dataset, focusing on the expression values from the creatine-related genes (Figure 6). Ten of the initial seventeen genes of the list had significative differential expression in the above dataset: GATM, SLC6A8, IGF1, AKT1, AKT3, PRPS1, CKB, SGK1, PSMD3, and PSMB5.

Notably, the expression levels of these genes can retrieve the exact clusterization of the experiment samples (TN and NSC samples) (Figure 6). Compared to the NSC samples, the TN samples have upregulated expression of transcript isoforms of the GATM and SLC6A8 genes. In addition, the average expression of IGF1 transcripts is also higher in TN. As previously described, GATM encodes the enzyme Glycine Amidinotransferase, which plays a crucial role in creatine biosynthesis, and IGF1 is related to creatine and amino acid metabolism. Furthermore, SLC6A8 codes the sodium- and chloride-dependent creatine transporter 1, responsible for creatine transport into cells against a concentration gradient. Recent studies have reported the transcriptional upregulation of this gene in tumoral cells under hypoxic conditions [46,47]. The authors of these studies concluded that increased creatine production or uptake promotes survival by maintaining redox homeostasis in hypoxic cells.

Moreover, reduced Reactive Oxygen Species (ROS) accumulation could activate AKT-ERK signaling, which protects the viability of hypoxic tumoral cells and explains the relationship between creatine accumulation and members of the AKT gene family. Nephrosclerosis patients may have reduced creatine production and accumulation in cells. Indeed, this compound is crucial to kidney functioning [10] and even more critical during gluconeogenesis cell metabolism to maintain energy levels when kidney cells rely on amino acids, fatty acid oxidation, and non-lactic pathways. Notably, the gradual reduction in tubular gluconeogenesis is a crucial feature of chronic kidney disease [48]. An uninterrupted and basal creatine supply could be why the TN samples had regular expression of these genes, which may reflect normal kidney functioning. However, further studies are necessary to elucidate if creatine supplementation would be an efficient adjuvant protocol to improve renal function under these conditions.

We also analyzed the dataset from Flechner et al. [49], which evaluated kidney biopsy expression profiles after transplant, unique to acute rejection (AR samples), dysfunction without rejection (NR samples), and well-functioning transplants (TX samples) (GDS724/GSE1563). For our analyses, we excluded the samples extracted from lymphocytes. Then, we evaluated the expression of the creatine-related genes of the TX, AR, and NR samples versus the control kidney biopsies. Not all of the genes were differentially expressed under our criteria (*p* < 0.05). This dataset is particularly interesting due to a recent study by Post et al. [10], who conducted a clinical trial on kidney transplant recipients, evaluating the creatine intake and endogenous production levels.

**Figure 7:**
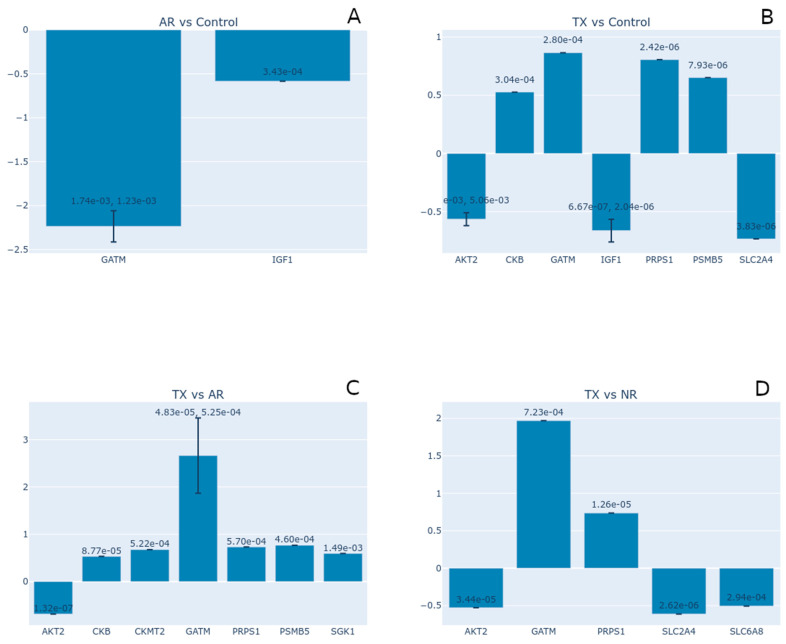
Expression analyses of creatine-related genes from transplanted patient kidney biopsies (dataset GDS724, [49]). (**A**) Acute rejection transplant (AR) versus control samples. (**B**) Well-functioning transplant (TX) versus control samples. (**C**) Well-functioning (TX) versus acute rejection transplant samples (AR). (**D**) Well-functioning (TX) versus dysfunctional non-rejected transplant samples (NR). The plot shows the log2 fold change for the genes that met the significance threshold in each comparison (*p* < 0.05). The error bars represent the standard deviation of a log2 fold change for genes with multiple measurements (distinct transcripts or probes for the same gene). This provides a measure of variability in the fold change estimate. Printed numbers in each bar describe the *p*-values.

Our first comparison was AR against control samples, where 1223 transcripts were differentially expressed. Among them, we observed differential expression for the creatine-related genes GATM and IGF1, both downregulated (Figure 7A). On the other hand, compared to the controls, the expression of TX samples presented DE of the genes AKT2, CKB, GATM, IGF1, PRPS1, PSMB5, and SLC2A4 (Figure 7B). We can observe that GATM has the most significant downregulation in AR versus the control, but this gene was upregulated in the TX vs. control comparison. In addition, IGF1 shows substantial changes in both comparisons, with smaller expression values in the TX vs. control comparison. We found no differential expression of the creatine-related genes in the NR samples compared to the controls.

We also compared the DE between the conditions. In the TX versus AR comparison, GATM shows the most significant upregulation, with a Log2 fold change around 2.5. CKB; PRPS1, CKMT2, PSMB5, and SGK1 are also upregulated to a lesser extent (log2 fold change between 0.5 and 0.8); and the gene AKT2 was slightly downregulated (Figure 7C). Compared to NR samples, the TX biopsies had increased expression of the genes GATM and PRPS1 and decreased expression of the genes AKT2, IGF1, and SLC6A8 (Figure 7D). Only IGF1 was upregulated in the comparison of the AR versus NR samples. These results demonstrate that the TX samples have induced expression of endogenous creatine production, marked by the increase in GATM compared to acute rejected transplant biopsies (AR) and dysfunctional but non-rejected samples (NR). This result might indicate that endogenous creatine production, or at least the activity of the GATM/AGAT enzyme, is the preferred pathway over creatine uptake. Post et al. [10] stated that kidney transplant recipients may be at risk of impaired creatine synthesis, and the results of the present analysis partially corroborate this.

**Figure 8:**
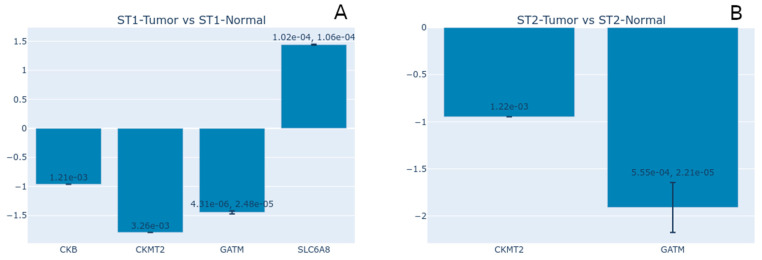
Expression analyses of the creatine-related gene transcriptional profile of two stages of clear cell renal cell carcinomas (cRCCs), paired with controls [51,52] (GEO Accession Number: GDS2880). (**A**) Stage I cRCC versus Stage I control samples. (**B**) Stage II cRCC versus Stage II control samples. The comparisons of Stage I versus Stage II, within normal and tumor samples, did not show differential expression of creatine-related genes. The plot shows the log2 fold change for the genes that met the significance threshold in each comparison (*p* < 0.05). The error bars represent the standard deviation of log2 fold change for genes with multiple measurements (distinct transcripts or probes for the same gene). This provides a measure of variability in the fold change estimate. Printed numbers in each bar describe the *p*-values.

Since there is no consensus on the role of creatine homeostasis in cancer cells [50], we decided to analyze the expression of the creatine-related genes in the dataset GDS2880 (series GSE6344), which evaluated the transcriptional profile of two stages of clear cell renal cell carcinomas (cRCCs) paired with controls [51,52]. The first analysis was the comparison of cRCCs versus control samples, both in Stage I, where we observed the differential expression of 4659 genes. Still, only four are creatine-related: CKB, CKMT2, GATM, and SLC6A8 (Figure 8A). The genes CKMT2 and GATM show the largest negative log2 fold changes, indicating significant downregulation in the tumor cells compared to the normal ones. On the other hand, the gene SLC6A8 exhibits substantial upregulation in the cRCC tissues. We also compared the Stage II cRCC samples versus their paired controls. In this analysis, we also found a strong downregulation of the genes GATM and CKMT2 in the cRCC samples. These results indicate that cRCC tissues have a severely affected endogenous creatine production. The higher expression of SLC6A8, especially during Stage I, might be a mechanism to increase the intracellular levels of this compound and maintain tumor viability [47] or to compensate for lower endogenous creatine synthesis to support kidney function.

It is interesting to note that the cRCC samples have downregulated GATM expression in both stages tested. The enzyme (AGAT) coded by this gene is the rate-limiting step of endogenous creatine biosynthesis [53]. Several previous studies have discussed renal GATM expression and AGAT activity in the context of kidney creatine contribution to total body guanidinoacetate (GAA) synthesis (approximately 20%). However, recent studies have related kidney GATM expression to renal function and homeostasis maintenance, where the capacity of GAA production decreases with increased states of CKD, becoming virtually nonexistent in dialysis patients [9]. On the other side of the spectrum, several mutations in GATM result in known genetic inborn diseases linked to creatine synthesis deficiency, like Cerebral Creatine Synthesis Deficiency 3 (CCDS3), and to altered phenotypes, like renal Fanconi syndrome and progressive kidney failure [54], and in dialysis-independent CKD patients [55]. Our analysis also reveals that downregulated GATM expression also occurs in cRCC samples, implying that increased creatine production, or at least GAA, may not be a strategy for this tumor type’s survival, especially during Stage II of the disease. It could also imply that regular GATM expression is essential for human kidney function.

**Figure 9:**
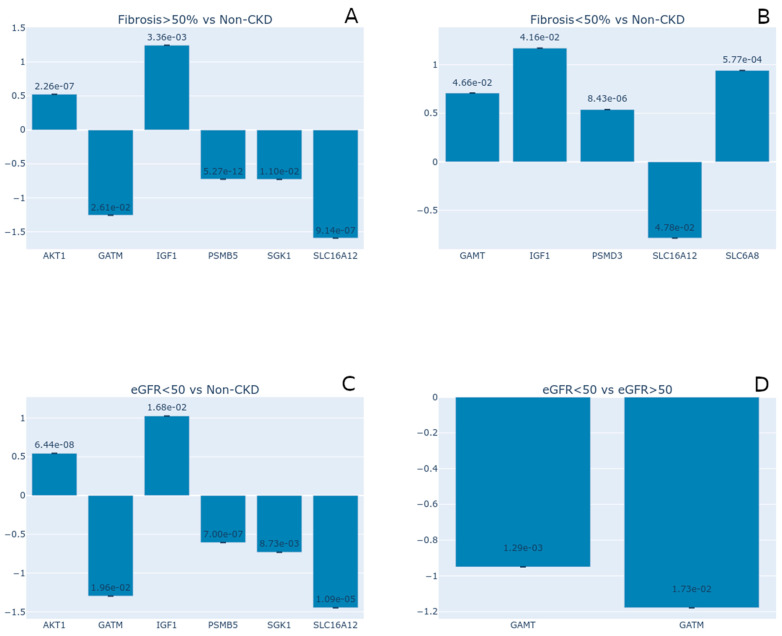
Expression analyses of creatine-related gene transcriptional profile from kidney fibrosis biopsies (GEO Accession Number: GSE137570). (**A**) Samples with tubulointerstitial fibrosis above 50% (fibrosis > 50%) versus non-chronic kidney disease (non-CKD) ones. (**B**) Tubulointerstitial fibrosis below 50% (fibrosis < 50%) versus non-CKD samples. (**C**) Samples with eGFR lower than 50 (eGFR < 50) versus non-CKD samples. (**D**) Comparisons between eGFR < 50 and eGFR > 50 samples. The plot shows the Log_2_ fold change for the genes that met the significance threshold in each comparison (*p* < 0.05). Printed numbers in each bar describe the *p*-values.

To better understand and confirm these observations, we analyzed the data of the RNA-Seq transcriptome profiling of chronic kidney disease from the series GSE137570 (BioProject: PRJNA565950; SRA: SRP222033). This data series provides clinical/morphological parameters for CKD samples, such as the estimated glomerular filtration rate (eGFR) and the tubulointerstitial fibrosis percentual of kidney biopsies. Using these parameters, we performed four different analyses. The first one was the general comparison of fibrosis samples versus non-chronic kidney disease tissue biopsies (non-CKD), with fibrosis samples having different values of % tubulointerstitial fibrosis, which ranged from 0 to 77.5%. So, we separated the samples into two additional groups with tubulointerstitial fibrosis above and below 50% (fibrosis > 50% and fibrosis < 50%, respectively). Four creatine-related genes presented downregulation: GATM, PSMB5, SGK1, and SLC6A12. Meanwhile, while AKT1 and IGF-1 were upregulated when comparing fibrosis > 50% to the non-CKD samples (Figure 9A). We also observed similar IGF-1 expression values when comparing fibrosis < 50% to the non-CKD samples (Figure 9B). The genes GAMT, SLC6A8, and PSMD3 showed significant upregulation in the 50% > fibrosis group (Figure 9C).

The fibrotic renal tissues from this cohort could have increased IGF1 due to insulin resistance or the first stages of DKD, or its upregulation could be linked to kidney hypertrophy, a known indicator of DKD, as previously demonstrated in animal models [38]. The action of this hormone-like molecule is to induce glucose consumption, inducing glycolytic enzymes, which, in turn, could lead to creatine synthesis. Renal tissues require high energy levels, and during CKD, this tissue has partial loss of fatty acid oxidation and an impaired capacity to produce glucose due to the loss of gluconeogenesis and an increase in glycolysis [10]. However, we did not observe differential expression of the GATM gene, which is the committing step for the pathway of this compound synthesis. Perhaps the GATM expression levels were close to the ones observed in the normal samples, maintaining GAA intracellular levels. GAA is toxic to the cell at high concentrations. Therefore, renal samples with lower than 50% tubulointerstitial fibrosis could upregulate the GAMT gene to metabolize GAA excess, leading to creatine synthesis. The significant upregulation of the gene PSMD3, a proteasome protease, might provide amino acids as substrates for this pathway and not provide carbon skeletons to the gluconeogenesis pathway, which would be reduced under the action of IGF-1 [38]. Since we also observed an upregulation of the creatine transporter gene (SLC6A8) in these samples, creatine must be related to renal function maintenance or energy supply. As fibrosis increases, the decreased expression of the monocarboxylic acid transporter, SLC6A12, may be linked to renal medulla osmoregulation. This transporter is crucial to the control of betaine accumulation [56].

We also categorized the samples from this study by their eGFR values, using a threshold of 50. We know this value is not a dividing point between the presence and absence of renal disease. The eGFR is indicative, calculated from the plasma creatinine levels, and, though related, is not equal to the actual glomerular filtration rate (GFR) [50]. The usual range used to indicate normal kidney function is above 90; values between 60 and 89 may indicate early-stage kidney disease; values between 15 and 59 may mean kidney disease; and values below 15 indicate kidney failure (National Institute of Diabetes and Digestive and Kidney diseases and National Kidney Foundation guidelines). However, we chose a lower threshold because gene expression alterations commonly occur before observable changes in clinical and morphological parameters. So, eGFR values indicative of early-stage renal disease may not imply the intense remodeling of gene expression, especially that related to energy production shifts.

Interestingly, the kidney biopsy samples with eGFR values below 50 (eGFR < 50) displayed similar differential expression of creatine-related genes to that in the samples with higher fibrosis percentages (fibrosis > 50%) (Figure 9A,C), which included the same DE genes. In addition, the samples with a lower eGFR (eGFR < 50) presented altered expression levels of the genes GATM and SLC16A12 (downregulated) and the genes IGF1 and AKT1 (upregulated) (Figure 9C). These observations demonstrate that samples with an eGFR < 50 have impaired endogenous creatine synthesis. Also, a previous study associated increased serum IGF1 levels with low eGFR rates [57]. From our gene list, only the GATM and GAMT genes were significantly downregulated when we compared the gene expression of the eGFR < 50 versus the eGFR > 50 samples (Figure 9D). These observations mean that there might be a relationship between the eGFR values and endogenous creatine production. These findings also corroborate the previous suggestions of [50,58] that with impaired endogenous creatine production in chronically and dialysis-dependent kidney disease patients, creatine becomes an essential nutrient, and these patients may require creatine supplementation. However, carefully evaluating the patient’s diet and creatine ingestion/supplementation is imperative to maintain kidney function and homeostasis.

Our creatine-related gene expression evaluation of the available transcriptomic data provides additional support for previous studies, but it is not without limitations. The case–control comparisons could be more complex because gene expression overlaps between different tested case conditions could occur. In addition, no information on the creatine content or consumption of the samples’ donors is available, which could directly impact our evaluations. Finally, the studies used experimental designs, which may need to be more suited to our questions. Therefore, we emphasize the need for specific studies with human or animal models that could address the questions raised here and confirm the central role of creatine homeostasis in kidney function.

### 3.6. Future Directions

Creatine supplementation still warrants a deeper understanding of its mechanisms of action, potential therapeutic applications in diseases, and its association with nephropathies. For instance, the potential elevation in serum creatinine levels resulting from the spontaneous conversion of creatine to creatinine was among the factors that led to scrutiny and questioning of the sale of creatine supplements in certain countries in the past. This appears to continue influencing new studies, as there is still hesitation in exploring the effects of creatine supplementation on nephropathies. Due to the spontaneous conversion of creatine to creatinine, serum creatinine should not be solely relied upon as a biomarker of renal function post-creatine supplementation. In such instances, elevated creatinine levels stem from increased creatine levels rather than renal dysfunction, resulting in a false positive indication of kidney injury. Other markers such as serum/urinary urea, cystatin-C, imaging, and molecular tests [59] should be considered in future studies to enhance the accuracy of renal function evaluation, whether in experimental or clinical settings.

In the case of diabetic nephropathy (DN), there are currently no studies confirming the effects of creatine supplementation in patients with this condition, only animal model studies [25]. However, like CKD and RT, some hypotheses suggest a potential positive outcome with creatine. Creatine supplementation might enhance renal function in DN patients by increasing glucose uptake through GLUT-4 translocation to the sarcolemma, as observed in type 2 diabetes patients [60]. However, the mechanism by which creatine supplementation increases GLUT-4 translocation has not been fully elucidated. The findings of Alves et al. [61] suggested that the increased expression of AMPK-α could explain the increased translocation of GLUT-4 to the cell membrane, but the increased expression of AMPK-α did not show statistical significance between the groups in this study. Therefore, this mechanism requires further investigation.

In a previous study, we observed that creatine supplementation did not induce significant changes in the renal morphology of diabetic animals [25]. Additionally, we found a hypoglycemic effect of creatine supplementation in this model. Based on the findings of Gualano et al. [60], the hypoglycemic effect we observed may have occurred due to the increased expression of GLUT-4 and unconventional myosin-Ic, proteins that could enhance glucose uptake.

In addition, several recent studies have suggested that the balance between creatine synthesis and uptake in renal tissues is directly linked to kidney function. Several abnormal kidney conditions induce the differential expression of creatine-related genes, especially GATM, GAMT, SLC6A8, AKTs, and IGF1. Further studies with specific experimental designs need to address whether the expression levels of these genes could be used as molecular markers of creatine homeostasis and kidney function.

## 4. Conclusions

The bioinformatics approach of this study indicated that genes such as GATM, GAMT, SLC6A8, and IGF1 are central to creatine metabolism, highlighting potential molecular mechanisms that may influence both kidney health and dysfunction. Our evaluation of the available transcriptomic data also gives new insights into the discussion about creatine’s role and its supplementation effects in kidney disease. The results also point to a complex interaction between creatine and metabolic pathways related to glycolysis, amino acid transport, and kinase regulation.

Although the study provided new insights, it also revealed significant gaps in the literature and in the available transcriptomic datasets, particularly regarding the effects of creatine supplementation in individuals with different types of kidney diseases. Therefore, future studies should focus on specific experimental models that directly assess the impacts of creatine supplementation, with an emphasis on its implications for kidney function and potential therapeutic applications.

These findings underscore the need to evaluate creatine supplementation in a personalized manner, considering the particularities of each renal condition, and suggest clinical potential that warrants further exploration.

## Supplementary Materials

The following supporting information can be downloaded at: https://www.mdpi.com/article/10.3390/nu17040651/s1, Figure S1: Expression of creatine-related genes and the kinases retrieved from gene enrichment analysis against kinase-specific databases. Obtained from the GTEx Portal (https://gtexportal.org). (TPM—Transcripts per million bases). Table S1: Protein kinases directly and indirectly related to the creatine-related genes according to our search criteria. Gene short descriptions were retrieved from GeneCards database.

## List of Abbreviations

ADP	Adenosine diphosphate
AGAT	Arginine:Glycine Amidinotransferase deficiency syndrome
AMPK-α	AMP-activated protein kinase alpha
AR	Acute rejection transplant
AOS	Active oxygen species
ATK1	V-akt murine thymoma viral oncogene homolog 1
ATK2	V-akt murine thymoma viral oncogene homolog 2
ATK3	V-akt murine thymoma viral oncogene homolog 3
ATP	Adenosine triphosphate
CK	Creatine kinase
CKB	Creatine kinase B
CKD	Chronic kidney disease
CKM	Creatine kinase, muscle
CKMT1A	Creatine kinase, mitochondrial 1A
CKMT1B	Creatine kinase, mitochondrial 1B
CTD	Comparative Toxicogenomics Database
DE	Differential expression
DN	Diabetic nephropathy
ERK1/2	Extracellular signal-regulated kinase ½
GAA	Guanidinoacetate
GAMT	Guanidinoacetate methyltransferase
GEO	Gene Expression Omnibus
GEA	Gene enrichment analysis
GLUT4	Glucose transporter type 4
GO	Gene Ontologies
GTEx	Genotype-Tissue Expression
HIV	Human Immunodeficiency Virus
IGF1	Insulin-like growth factor 1
KEGG	Kyoto Encyclopedia of Genes and Genomes
MCT12	Monocarboxylate Transporter 12
MESH	Medical Subject Headings
MtCK	Mitochondrial creatine kinase
MYO1C	Myosin IC
NSC	Nephrosclerosis
PFKFB3	6-phosphofructo-2-kinase/fructose-2,6-biphosphatase 3
PKM2	Pyruvate kinase isozymes M2
PPI	Protein–protein interaction
PSMB5	Proteasome subunit beta type-5
PSMD3	26S proteasome non-ATPase regulatory subunit 3
PTEN	Phosphatase and tensin homologue
RT	Renal transplant
SGK1	Serine/threonine-protein kinase 1
SLC2A4	Solute Carrier Family 2 Member 4
SLC6A8	Solute Carrier Family 6 Member 8
SLC6A12	Solute Carrier Family 6 Member 12
TN	Tumor nephrectomy
TP53	Tumor protein p53
TPM	Transcripts per million
TX	Well-functioning transplant
