# Supplementary material for: Assessing Creatine-Related Gene Expression in Kidney Disease: Can Available Data Give Insights into an Old Discussion?
Source: Nutrients. 2025 Feb 12;17(4):651. doi: 10.3390/nu17040651 (PMC11858045; doi:10.3390/nu17040651)

**Supplementary Figure S1.** Expression of creatine-related genes and the kinases retrieved from gene enrichment analysis against kinase-specific databases. Obtained from the GTEx Portal (https://gtexportal.org). (TPM - Transcripts per million bases).


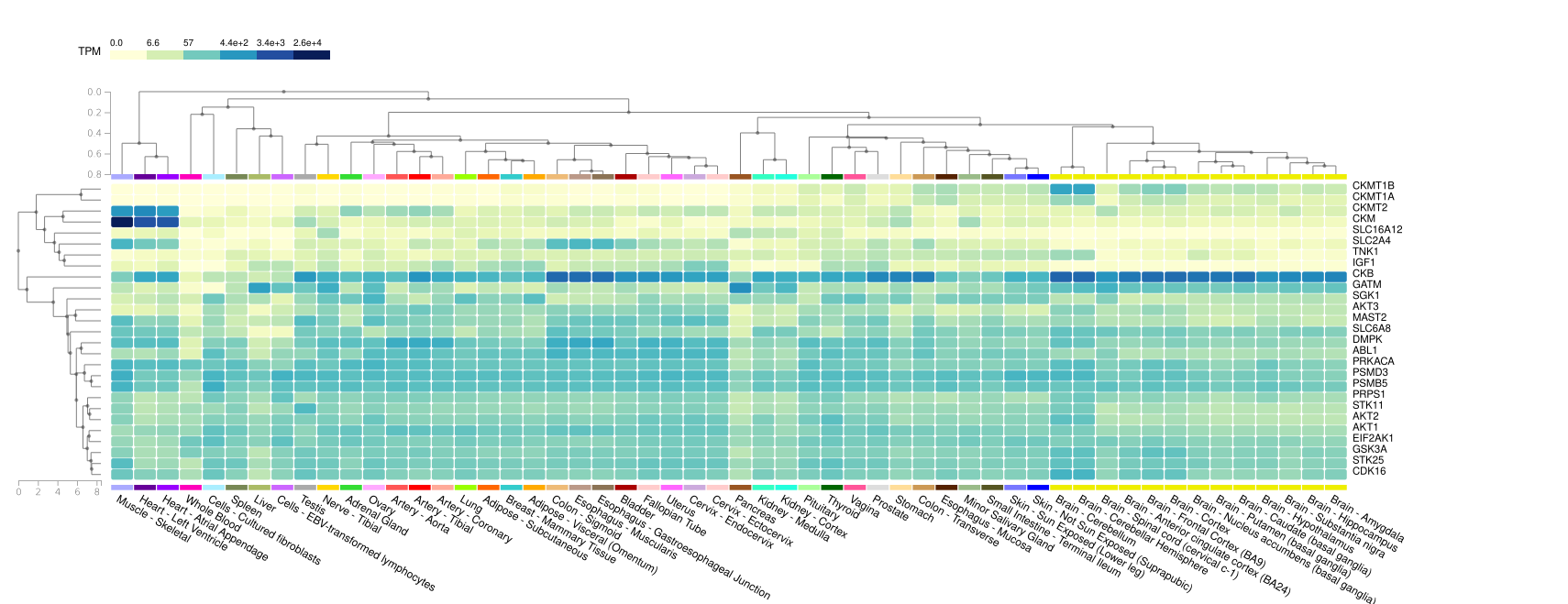

Supplement: Supplementary file 1 [file nutrients-17-00651-s001.zip › Supplementary Figure S1.docx]
