# Supplementary material for: Assessing Creatine-Related Gene Expression in Kidney Disease: Can Available Data Give Insights into an Old Discussion?
Source: Nutrients. 2025 Feb 12;17(4):651. doi: 10.3390/nu17040651 (PMC11858045; doi:10.3390/nu17040651)
Supplement: Supplementary file 1 [file nutrients-17-00651-s001.zip › Supplementary Table S1.docx]

**Supplementary Table S1.** Protein kinases directly and indirectly related to the creatine-related genes according to our search criteria. Gene short descriptions were retrieved from GeneCards database.

| **Kinase Gene Symbol** | **Short description (Source: GeneCards)** |
| --- | --- |
| CAMKK2 | Calcium/calmodulin-dependent protein kinase kinase 2, involved in various signaling pathways. |
| MAP2K2 | Mitogen-activated protein kinase kinase 2, a key component in the MAPK/ERK pathway. |
| VRK1 | Serine/threonine-protein kinase VRK1, involved in cell cycle and nuclear envelope formation. |
| PDK4 | Pyruvate dehydrogenase kinase isozyme 4, regulating glucose and fatty acid metabolism. |
| PLK4 | Polo-like kinase 4, a regulator of centriole duplication in cell cycle progression. |
| PDK1 | Pyruvate dehydrogenase kinase 1, regulating glucose metabolism by phosphorylating pyruvate dehydrogenase. |
| DMPK | Myotonic dystrophy protein kinase, involved in muscle, heart, and brain function. |
| AAK1 | AP2 associated kinase 1, involved in clathrin-mediated endocytosis. |
| TBK1 | TANK-binding kinase 1, central in innate immune response and inflammation. |
| GAK | Cyclin G-associated kinase, involved in cell cycle regulation and clathrin-mediated endocytosis |
| NEK7 | Serine/threonine-protein kinase involved in mitotic cell cycle progression and cytokinesis. |
| PBK | Serine/threonine-protein kinase involved in mitotic cell cycle and DNA damage response. |
| BMP2K | Kinase implicated in bone morphogenic protein signaling and osteoblast differentiation. |
| MASTL | Kinase that regulates mitosis entry and maintenance which is involved in cell cycle checkpoint recovery. |
| VRK2 | Kinase involved in cell cycle regulation and neuronal apoptosis. |
| STK25 | Kinase that regulates stress response and cell migration. |
| CDK16 | Kinase involved in vesicle-mediated transport and exocytosis. |
| MAST2 | Kinase that regulates spermatid differentiation and interleukin-12 production. |
| PRKACA | Catalytic subunit of protein kinase A, involved in various cellular processes including metabolism and memory. |
| ABL1 | Tyrosine-protein kinase involved in cell differentiation, division, adhesion, and stress response. |
| STK11 | Serine/threonine kinase 11, also known as liver kinase B1 (LKB1), which regulates cell polarity and functions as a tumor suppressor. |
| TNK1 | Tyrosine kinase non-receptor 1, involved in negative regulation of cell growth and acting as a tumor suppressor. |
| EIF2AK1 | Eukaryotic translation initiation factor 2-alpha kinase 1, which plays a role in the cellular response to stress by phosphorylating the alpha subunit of the eukaryotic translation initiation factor 2 (eIF2). |
| GSK3A | Glycogen synthase kinase 3 alpha, involved in energy metabolism, neuronal cell development, and body pattern formation. |

1. Stelzer, G.; Rosen, N.; Plaschkes, I.; Zimmerman, S.; Twik, M.; Fishilevich, S.; Stein, T.I.; Nudel, R.; Lieder, I.; Mazor, Y.; et al. The GeneCards Suite: From Gene Data Mining to Disease Genome Sequence Analyses. Curr. Protoc. Bioinform. 2016, 54, 1–30
